# Mind in motion: patients’ experiences with group-based physical activity in psychiatric treatment- a mixed-methods study

**DOI:** 10.1186/s12888-026-08117-7

**Published:** 2026-04-26

**Authors:** Siri Elicabeth Hansson, Frank Eirik Abrahamsen, Geir Haakstad, Christina Gjestvang, Rannei Johanne Grimsmo Jørgensen, Wendy J. Brown, Lene Annette Hagen Haakstad

**Affiliations:** 1 Lovisenberg Diaconal Hospital, Oslo, Norway; 2 Department of Sport and Social Sciences, Norwegian School of Sports Sciences, Oslo, Norway; 3 Sports Pedagogue, Diakonhjemmet Hospital, Department of Adult Psychiatry, Vinderen, Oslo Norway; 4 Department of Sports Medicine, Norwegian School of Sports Sciences, Oslo, Norway; 5 Faculty of Health Sciences and Medicine, Bond University, Gold Coast, Australia; 6 Faculty of Health, Medicine and Behavioural Sciences, University of Queensland, Brisbane, Australia

**Keywords:** Mixed-methods, Patients’ experiences, Physical activity, Psychiatric treatment, Severe mental illness

## Abstract

**Background:**

Individuals with severe mental illness face significant health challenges, such as cardiovascular diseases and social isolation. Physical activity is increasingly recognized as an integral part of psychiatric treatment, with evidence showing it can prevent and manage somatic diseases, enhance mental health, reduce premature mortality, and support social inclusion. In Oslo, Norway, group-based physical activity is offered as part of routine care at Diakonhjemmet Hospital. Participants attended one to four sessions per week, including endurance- and strength-based activities such as hiking, swimming, and strength training. In this study, we aimed to explore how adults with severe mental illness experienced this group-based physical activity program during psychiatric treatment.

**Methods:**

Participants were receiving psychiatric treatment for severe mental illness, including schizophrenia spectrum disorders and bipolar disorder. This qualitative-driven mixed-methods study combined eight in-depth, semi-structured interviews with questionnaire data from 33 adults, including the interview participants. Three main areas were explored: (1) holistic health benefits (2), addressing barriers, and (3) transition and continuity. Data analyses were organised around the three predefined study areas, using reflexive thematic analysis of the interview data and descriptive statistics from the questionnaire. Quantitative results were integrated at the thematic level to enrich interpretation and understanding.

**Results:**

Within each study area, two-three themes were constructed. Combined findings from both methods showed that participants experienced broad health improvements, including fostering well-being, social connectedness, and structured daily routines. Perceived barriers were related to medication side effects, fatigue, and fluctuating motivation. Support from knowledgeable and enthusiastic activity leaders was essential in helping participants overcome barriers and stay active. While most participants intended to remain active after treatment, limited follow-up support and access to similar community programs hindered the prospect of continued engagement.

**Conclusions:**

Participants perceived that tailored and well-led physical activity during treatment contributed to improvements in health and social connectedness. However, long-term maintenance was perceived to be hindered by limited access to follow-up programs, highlighting the need for better post-treatment support. These findings underscore the importance of expanding access to physical activity programs within real-world psychiatric care, to ensure that individuals with severe mental illness have genuine opportunities to establish physical activity as a routine part of daily life.

**Supplementary Information:**

The online version contains supplementary material available at 10.1186/s12888-026-08117-7.

## Background

Mental disorders represent a significant global health challenge, affecting nearly one billion people [1]. Individuals with severe mental illness, including schizophrenia spectrum disorders, bipolar disorder, and major depressive disorder, have a reduced life expectancy of 10–20 years [2]. This disparity is primarily due to lifestyle-related factors such as poor diet, smoking, alcohol use, inadequate sleep, and low levels of physical activity, combined with side effects from antipsychotic medications [1, 3].

Physical activity is increasingly recognized as an effective yet underused component of mental health care [4]. Evidence shows that regular physical activity can reduce symptoms of depression and anxiety, prevent somatic diseases, enhance physical health and social functioning, and improve quality of life [5, 6]. National and international guidelines recommend incorporating physical activity into standard psychiatric treatment [7–9]. However, individuals with severe mental illness face distinct barriers to being active, including unstable or fluctuating symptoms, medication side effects, and stigma [10]. Some psychiatric institutions have employed physical activity professionals to deliver tailored, holistic interventions that may help to overcome these barriers [11]. However, despite strong evidence and policy support, physical activity remains underprioritized in routine clinical practice [6].

Previous studies have highlighted the benefits of exercise in psychiatric settings, such as improved symptoms, social interaction, and daily structure [12–15]. While some of this work has included patient perspectives, much of the existing literature has focused on staff attitudes and perspectives. Moreover, few studies have combined qualitative and survey data to explore patients’ lived experiences, perceived barriers, and the factors that support long-term engagement in a structured physical activity program within outpatient mental health care services. Understanding how patients engage in and sustain physical activity is crucial to effective implementation and to reducing the 10–20-year mortality gap associated with severe mental illness [16].

This study was conducted at Diakonhjemmet Hospital in Oslo, Norway, where group-based physical activity is integrated as part of routine psychiatric treatment. Participants take part in one to four sessions per week, including endurance- and strength-based activities such as hiking, swimming, and strength training, adapted to seasonal conditions and individual capacity. Structured and systematically supported physical activity programs of this kind within Norwegian mental health care services appear understudied [16]. To address this gap, we employed a mixed-methods approach to provide a more comprehensive, nuanced, and context-specific understanding of patients’ experiences. This may support further development and implementation of physical activity programs within Norwegian mental health care services.

The aim of this mixed-methods study was to explore how patients with severe mental illness experience participation in a group-based physical activity program, focusing on: (1) perceived health benefits; (2) barriers and strategies for managing these; and (3) factors supporting continued engagement beyond treatment.

## Methods

This study is part of the Mind in Motion (MiM) project, a collaboration between the Norwegian School of Sport Sciences (NIH) and Diakonhjemmet Hospital. The aim of MiM is to examine the role of physical activity in psychiatric treatment through two complementary sub-studies: part A (the present study) focuses on patients’ perspectives, while part B focuses on healthcare professionals’ perspectives.

To explore patient perspectives in depth, in part A, we employed a convergent mixed-methods design, combining semi-structured interviews and data from questionnaires. The two methods addressed the same core topics: perceived benefits, barriers, and sustainability of physical activity. Data were analyzed separately and integrated through side-by-side comparison [17]. The qualitative-driven approach allowed in-depth insights from selected active program participants to be complemented and contextualized by quantitative data from a larger group of occasional program participants. The study adheres to COREQ and GRAMMS reporting guidelines.

### Setting

The Department of Adult Psychiatric Care at Diakonhjemmet Hospital is a specialized outpatient clinic for patients with severe mental illness, with or without co-occurring substance use. Most patients receive follow-up care in the specialist mental health care services for periods ranging from several months to many years, depending on their ability to benefit from treatment and their progress over time. Approximately 350 patients are registered with the outpatient clinic; most are between 18 and 65 years of age, after which they are referred to geriatric psychiatric services.

In this department, a multidisciplinary resource team organizes a voluntary physical activity program which is integrated into routine psychiatric care. The program is led by a sports pedagogue and an occupational therapist who offer a range of group-based indoor and outdoor activities throughout the year, including seasonal social events and trips, designed to foster shared mastery experiences. The weekly activity program consists of one group-based activity, at set times during working hours each weekday. Patients are eligible for the activity program if they are currently associated with the specialized outpatient clinic and obtain access through a referral from a healthcare professional at the clinic. Because referral alone does not ensure engagement, the activity leaders play an active role in motivating and following up with patients, as those who initially agree to participate often require ongoing support before they feel ready to attend regularly. To support engagement, the sports pedagogue emphasizes individualized adaptation. This includes regular motivational follow-up, tailoring activities to each participant’s needs and functional level, and organizing sessions at easily accessible locations, to reduce practical barriers.

### Researcher’s positioning

The first author (SEH) is currently a PhD fellow but was a master’s student in Sports Medicine at the Norwegian School of Sports Science (NIH) during data collection. She designed the study together with LAHH, CG, and GH, collected all the data (with GH assisting on the surveys), analyzed the data, and wrote the initial draft of this paper. One year prior to the study, she completed a four-week internship with the outpatient clinic’s resource team, as part of her bachelor’s program at NIH. However, she was not involved in clinical treatment or in delivering the physical activity program during the study period. SEH conducted the in-depth interviews, and her prior familiarity with the setting and patient group was recognized, with ongoing reflexive considerations to enhance transparency and reduce potential bias.

### Participants

Participants in the group-based physical activity program had diagnoses of schizophrenia spectrum disorders, bipolar disorder, and/or major depressive disorder. Recruitment was carried out in collaboration with health care professionals at Diakonhjemmet Hospital. GH, the sports pedagogue responsible for the physical activity program but not involved in participants’ clinical treatment, first identified eligible patients, informed them about the study, and collected contact information from those interested. The master’s student (SEH) then contacted potential participants to provide standardized information, answer questions, obtain informed consent, and schedule interviews. GH provided standardized information and obtained informed consent for the questionnaire component.

Eligibility for the study was assessed to ensure that participants met the inclusion criteria and were able to provide informed consent and participate in an interview independently. The inclusion criteria for both the in-depth interviews and questionnaire were: ≥18 years of age, capacity to provide informed consent, participation in the weekly activity program at least once per week during the previous four weeks, and ongoing treatment for severe mental illness.

Two samples were recruited:

*Active program participants* participated in both interviews and questionnaires, conducted between March and May 2024. Of twelve eligible and approached, eight (66.7%) consented and were included, comprising five women and three men. Reasons for non-participation included illness, anxiety, or lack of time. This sample was deemed sufficient for the qualitative interviews as data saturation was reached with no new themes emerging. Hence, different dimensions and meanings were well captured [18].

*Occasional program participants* completed only the questionnaire, having attended ≥ 1 session/week in any four weeks during the past six months. Of 36 eligible patients, 25 (69.4%) were included between March 2024 and February 2025, with eleven excluded due to illness (*n* = 6) or not meeting the inclusion criteria of having attended > 1 session/week in any four weeks during the past six months (*n* = 5). Hence, the total questionnaire sample was 33 (8 active + 25 occasional participants). There were no dropouts in either sample.

### Data collection

#### Interviews

Semi-structured interviews were conducted at NIH or Diakonhjemmet Hospital by SEH and lasted on average 40 min. A piloted interview guide (see Additional file 1) with open-ended questions ensured consistency. Reflexive field notes were taken, and interviewees completed the questionnaire immediately before their interview. The interviews were audio-recorded and transcribed verbatim using Nettskjema-diktafon, with transcripts manually checked for accuracy by the same researcher. With one exception, interviews were held immediately after a scheduled activity session.

#### Questionnaires

Questionnaires were distributed by GH in coordination with participants’ schedules between March 2024 and February 2025. The 10-minute paper-based questionnaire (see Additional file 2) was based on *existing validated questionnaires* with items covering demographic characteristics, psychological well-being on a scale from 0= “worst possible mental well-being” to 10= “best possible mental well-being” (*MHQoL)* [19], non-communicable diseases, physical activity level and type, perceived benefits and barriers (*Kan3- Mapping of Physical Activity Among Adults and Older Adults 2020–22)* [21], and motives *(EMI-2*) [20], supplemented by adapted items from Ussher et al. (2007) [22], Gjestvang et al. (2020) [23], and Haakstad et al. (2017) [24]. Ratings of positive effects on physical and mental health were assessed on a scale of 1–10 (1 = “no positive effect”, 10 = “very positive effect”). The questionnaire primarily consisted of multiple-choice items with optional text fields for elaboration on motives and barriers to participation. None of the free-text responses offered information beyond the predefined answer options. Age, height, and weight were reported using open-ended responses.

### Data analysis

An interpretive, pragmatic mixed-methods approach was applied [25, 26], guided by the salutogenic health model [27] as a theoretical framework. This framework views health as a continuum, emphasizing factors that promote well-being rather than those that cause disease [28]. Additionally, abductive analysis incorporated established theory while allowing for deviations, combining inductive and deductive reasoning to develop theory [29].

The analyses were structured around three predefined study areas (holistic health benefits, addressing barriers, and transition and continuity) derived from the research questions. Within each focus, themes were analytically constructed from the qualitative interview material, with quantitative findings integrated at the thematic level to support interpretation. All analyses were conducted by the first author (SEH) and later discussed and refined with the interdisciplinary research group.

#### Qualitative analysis

Interview data were analyzed in MAXQDA24 using Braun and Clarke’s (2006) thematic analysis [30, 31], which generated 12 initial codes that were refined into two or three themes within each of the three study areas. This reflexive and flexible process allowed movement between analytic phases, enabling iterative return to earlier stages to reflect on and refine codes and themes so that participants’ meanings were adequately represented. Detailed interpretations, supported by participant quotations, are presented in the results.

#### Quantitative analysis

Questionnaire data were manually entered into IBM SPSS Statistics by SEH and double-checked for errors by a second researcher (RJGJ). Descriptive statistics and univariate analyses were used to summarize data in each of the project’s main focus areas. No composite scores were used.

#### Data integration

Findings from the interviews and surveys were merged through a side-by-side comparison to identify convergences and divergences and enrich interpretation as encouraged by Guetterman, Fetters & Creswell (2015) [32].

### Ethical considerations

This study was approved by the Regional Committees for Medical and Health Research Ethics in Norway (REK) (ref. 701157), the Ethical Review Board at NIH (ref. 130324), and the Norwegian Agency for Shared Services in Education and Research (Sikt) (ref. 316538). Two former patients from Diakonhjemmet Hospital contributed to the study design as user representatives. Participation was voluntary, written informed consent was obtained, and data were non-identifiable and securely stored by the Information Technology Department at the Norwegian School of Sport Sciences, in accordance with Norwegian regulations. No financial compensation was provided. To minimize any sense of pressure to participate, information about the study was provided by the first author (SEH) rather than any treating clinician. Voluntary participation was explicitly emphasized, and participants were informed that choosing to participate or not would have no consequences for their treatment.

Furthermore, data were de-identified, and participants were informed that their treatment team would not have access to their responses. Hospital personnel verified study eligibility and were available to assist during and after the interviews to address potential distress. No adverse events occurred.

## Results

### Participants

Characteristics of the 33 participants are shown in Table 1. Most had completed higher education, were unemployed, and had a diagnosis of schizophrenia, with no comorbid conditions. On average, they participated in the physical activity program twice per week (Table 1).

More details of the interview sample are shown in Additional file 3. In this sample, more than half had schizophrenia spectrum disorder, and none were employed at the time. Two attended the physical activity program once a week, four twice a week, and two four times a week. The most common activities were hiking and swimming. Six participants were also physically active outside treatment, mainly through active commuting. To protect confidentiality, psychiatric diagnoses were not specified, fictional names were randomly assigned based on gender, and participants were placed in age categories.

**Table 1:** Participant characteristics for the total sample combining both methods (n = 33)

Characteristics	Total (*n* = 33)
Age (years) (mean (SD)) [range]	41.8 (11.7) [29–61]
BMI (kg/m^2^)^1^ (mean (SD)) [range]	27.9 (5.8) [20.5–41.4]
Work status (n (%))	
Unemployed/disabled Working < 50% Working > 50% Student/apprentice	26 (78.8) 3 (9.1) 2 (6.1) 2 (6.1)
Highest completed education^2^ (n (%))	
Primary education (< 10 years) Secondary education (11–13 years) Higher education (university/college) Other	7 (21.2) 9 (27.3) 14 (42.4) 3 (9.1)
Mental illness (n (%))	
Schizophrenia Did not wish to disclose Bipolar disorder Another form of psychosis Schizoaffective	13 (39.4) 7 (21.2) 5 (15.2) 5 (15.2) 3 (9.1)
Comorbid diseases (n (%))	
None Asthma Allergy Type 2 diabetes COPD^3^ Hypertension Cancer Eating disorder Rheumatic disorder	22 (66.7) 3 (9.1) 3 (9.1) 3 (9.1) 2 (6.1) 1 (3.0) 1 (3.0) 1 (3.0) 1 (3.0)
PA-level in treatment (days per week) (mean (SD)) [range]	2.0 (1.3) [1–4]
Mental well-being (0–10)^4^ (mean (SD)) [range]	7.0 (1.9) [3–10]

Note: SD= standard deviation, range= minimum-maximum, ^1^Body mass index (BMI) was calculated using Helsedirektoratet’s “KMI-kalkulator” (WHO overweight threshold: >25 kg/m^2^), ^2^International education classification systems, ^3^COPD= Chronic obstructive pulmonary disease, ^4^MHQoL 0–10 perceived mental well-being on the day of filling out the questionnaire (from 0 = worst possible mental well-being to 10 = best possible mental well-being). Mental illness categories reflect participants’ self-reported primary diagnosis and are therefore mutually exclusive. Comorbid diseases could be selected in multiple categories

### Integrated presentation of qualitative and quantitative findings

**Fig. 1:**
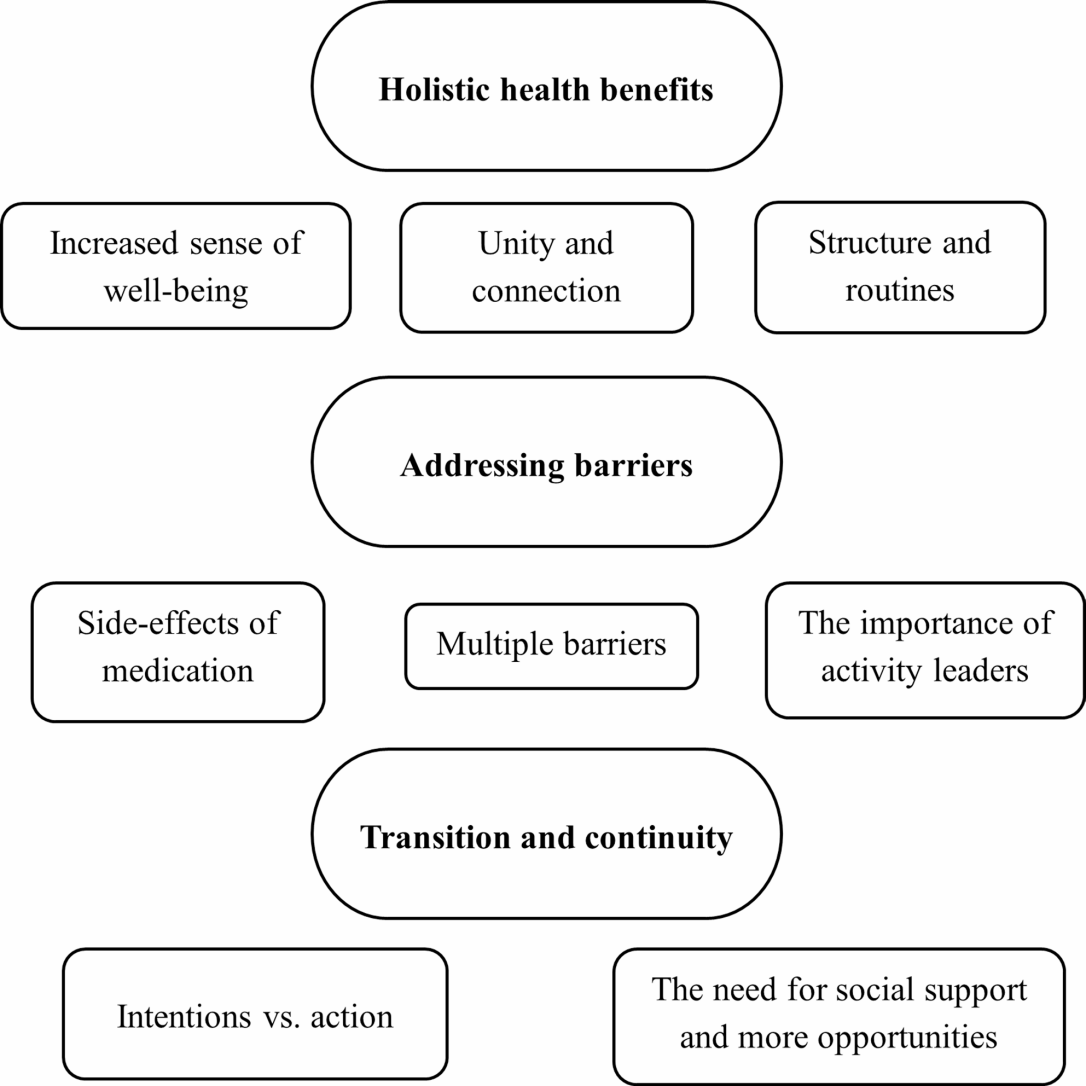
Overview of study areas and the analytically constructed themes. Illustration style inspired by McCall et al. (2023) [34]

Eight themes were constructed within the three predefined study areas (Fig. 1). These themes were integrated with corresponding quantitative results through side-by-side comparison to enrich interpretation and provide a more comprehensive understanding of participants’ experiences [32, 33].

### Holistic health benefits

The first theme, *increased sense of well-being*, captured how participation gave relief from symptoms and fostered a more positive outlook on life: *“I think it makes my life better. I feel like I’ve regained a part of myself somehow”* (Mary, 60s, attending twice a week). Several participants emphasized the restorative effects of being active outdoors: *“I enjoy nature. I let my mind wander and think about other things”* (John, 60s, attending twice a week). Others described the sense of accomplishment that followed after sessions. *“After the activity*,* it feels great to have completed it. It gives you a boost”* (Sophia, 40s, attending four times a week).

The second theme, *unity and connection*, illustrated how group sessions reduced feelings of isolation and supported a sense of belonging. One participant shared, *“I don’t feel lonely anymore (…) I feel like I’m part of something important”* (Daniel, 30s, attending four days a week). Others highlighted that being expected by peers increased commitment and motivation to attend.

The final theme, *structure and routines*, described how regular, scheduled activities brought rhythm and consistency to daily life: *“It feels worthwhile and constructive*,* having something meaningful to do during the day (…) That it resembles*,* I don’t know*,* a normal life”* (Anna, 30s, attending twice a week). Another emphasized the motivational effect of activities having fixed times: *“It helps to have an appointment at a set time*,* that way you’re more likely to stick to it”* (Eric, 30s, attending twice a week).

*Questionnaire data* (see Table S1 in Additional file 4 for more specific details) supported these qualitative findings. The majority of participants (90%) reported joining the activity program to “have a healthy body” and “get fresh air”, while 82% described social reasons for participation. Mean ratings of perceived positive effect of participation were 7.9 for physical health and 7.7 for mental health (on a scale from 1: “no positive effect” to 10: “very positive effect”). Eighteen participants (55%) had received encouragement to engage in the physical activity program from healthcare professionals at Diakonhjemmet Hospital, a topic that was less emphasized in the interviews.

### Addressing barriers

The first theme, *side-effects of medication*, illustrated how fatigue and weight gain reduced participants’ energy and motivation to be active. As Anna explained, *“Things can feel heavy*,* and it takes some time to get the engine running (…) if you take medication in the morning*,* you can feel sluggish”.* Others described weight gain as the main obstacle: *“The big obstacle for me is the weight gain (…)”* (Helen, 50s, attending once a week). Some participants suggested that dietary guidance could be integrated into the physical activity program to better address weight-related barriers.

The second theme concerned *multiple barriers* beyond medication, encompassing practical challenges, poor sleep, low motivation, and seasonal conditions. As John explained, *«As long as I have sleep problems*,* nothing really works”*. Others emphasized the importance of maintaining voluntary participation, as Anna noted: *“If it gets too strict or forced*,* you lose some of your motivation”*. A few participants also described feeling uneasy about participating in a group defined by illness, explaining that being classified as “sick” could reinforce stigma and affect their motivation to attend.

The final theme, *the importance of activity leaders*, described how their involvement helped participants overcome barriers. Mary shared, *“It’s easier because [the sports pedagogue] is a motivator.”* Their expertise and ability to create a sense of safety and cohesion were especially valued: *“I see them as*,* like*,* safe people (…) we feel like a team”* (Anna).

*The questionnaire data* (see Table S2 in Additional file 4 for more specific details) were consistent, showing strong alignment across methods. Nearly all participants (97%) agreed with the statement: “I feel supported by the activity leaders in the program” and reported missing an average of 0.9 sessions per month, mainly due to practical or motivational-related barriers. Furthermore, 70% believed that physical activity should be a permanent part of mental health care services.

### Transition and continuity

The first theme, *intentions vs. action*, reflected the gap between motivation and implementation. While most participants expressed a wish to stay active, the level of planning varied. Sophia explained: “*I believe the routine is already there (…)*,* I think if I just keep doing the same kind of exercise and sign up somewhere*,* it’ll be fine”.* Others were less certain, and Eric described the challenge of moving from structured activities in treatment to taking personal responsibility for maintenance: *“There are two mindsets: one is being in a hospital and doing activities*,* the other is bringing it with you when you’re finished.”*

The second theme, *need for social support and more opportunities*, showed that several participants perceived a lack of suitable community options: *“Maybe just spread the offer to more parts of the community. That would make it easier”* (Eric). Receiving a simple list of options was considered unhelpful: *“I ended up watching TV instead*,* and the list just ended up in a drawer”* (Daniel). Many emphasized social support as essential for maintaining physical activity after treatment: *“I’ll never do it alone. I’m pretty sure about that”* (Helen).

Consistent patterns emerged in the *questionnaire data* (see Table S3 in Additional file 4 for more specific details), where participants’ preferences closely aligned with the activities they had already engaged in during treatment (e.g., hiking, strength training, swimming). While in-depth interviews indicated limited awareness of community options, 63.6% of the questionnaire participants reported being aware of alternatives. A large majority (91%) also wished to be active with others in some form, reporting social support as a key factor for long-term engagement.

## Discussion

In this mixed-methods study, we explored how individuals with severe mental illness experienced a group-based physical activity program within real-world psychiatric treatment. The findings highlight that regular participation was associated with enhanced well-being, social connectedness, and the establishment of meaningful routines. Participants described physical activity as a source of empowerment, structure, and a sense of belonging, supported by quantitative data. Despite these benefits, several barriers were identified, including medication side effects and motivational challenges. The activity leaders emerged as key facilitators, fostering safety, motivation, and group cohesion. However, participants emphasized the need for ongoing social support and accessible community opportunities in order to maintain activity at the end of treatment.

### More than movement: physical activity as a foundation for holistic health

Participants viewed health holistically, perceiving the physical, mental, and social dimensions of the physical activity program as deeply interconnected. Physical activity was valued not only for its potential to alleviate symptoms but also for fostering structure, belonging, and personal growth. This holistic perspective echoes previous qualitative work showing that patients appreciate the universal therapeutic benefits as much as physical outcomes [35]. Unlike studies where appearance and fitness motives dominate [36], our qualitative findings showed that aesthetic goals were largely absent. This pattern was echoed in the questionnaire data, where participants experienced social and emotional benefits just as highly as physical health outcomes. This suggests that different aspects of health are closely linked and should be explored together in future research.

The program’s social dimension appeared particularly meaningful to participants. This finding from both methods is consistent with previous research showing that social interactions can feel more meaningful to participants than physical changes such as weight loss or muscle gain [37, 38]. The social environment itself may be just as important as the activity [39]. Therefore, interventions could benefit from focusing on patients’ values and overall well-being, rather than only on traditional physical outcomes.

The physical activity program also appeared to provide a predictable and meaningful routine, which may be especially valuable for individuals with severe mental illness who often face unemployment, isolation, and instability [35, 40]. This structure may have helped to reduce feelings of alienation and to foster a sense of normalcy in daily life, echoing the findings of Hargreaves et al. (2017) [41]. As previous research has indicated, physical activity can act as a life-structuring resource that fosters resilience, autonomy, and interpersonal connections [28]. Thus, participants’ perception of structured routine as a health benefit is particularly noteworthy and warrants further investigation. Given the complex etiology of severe mental illness, which is shaped by genetic, environmental, and social factors [42], these findings may support a salutogenic perspective of health-promoting resources, like routine and social connection, over pathology alone.

### Overcoming barriers: navigating physical activity in psychiatric treatment

Despite the perceived benefits of physical activity, participants encountered several fluctuating barriers, such as medication-related weight gain, fatigue, and practical challenges. Such barriers are well-documented in the literature [43] and were also reported by nearly half the participants in this study. Some participants suggested that dietary counselling could complement the physical activity program, echoing recent calls for more integrated lifestyle interventions [44]. These varied barriers illustrate the complexity of designing and implementing physical activity interventions in psychiatric care, where patients must constantly adapt to their current mental and physical state [10].

Activity leaders appeared to play a key role in helping participants overcome these barriers. Their encouragement, expertise, and adaptability seemed to foster both trust and motivation. This aligns with evidence suggesting that supportive relationships can enhance adherence, with some studies even comparing the activity leader’s therapeutic role to that of a psychotherapist [39, 45]. At the same time, interviewed participants highlighted the importance of autonomy, preferring voluntary over mandatory participation in exercise. Notably, in the questionnaire, participants agreed with the item stating that “physical activity should be a permanent component of treatment”. This difference illustrates how wording and context shape interpretation - “mandatory” can sound pressured, whereas “permanent” implies continuity [46]. Thus, interviewees may have responded more negatively because they associated “mandatory” with coercion, while questionnaire respondents may have viewed a “permanent part of care” as something positive, reliable, and available by choice. Participants may also support PA being embedded in mental health care because engagement can fluctuate during recovery, and because individuals with severe mental illness often feel safer in supported exercise settings than in public gyms [10]. These nuances underline the importance of precise language when designing and communicating about physical activity interventions in mental health care.

Barriers were not only individual but also systemic. Over half the questionnaire participants reported conflicting treatment appointments, a challenge not raised in interviews, demonstrating the added value of a mixed-methods approach. These observations call for stronger interdisciplinary coordination to position physical activity as a shared responsibility across mental health services [47]. Overall, these findings suggest that flexible, individualized, and co-designed programs may be more likely to improve participation and adherence [48, 49].

### Sustaining momentum: supporting physical activity after psychiatric treatment

Maintaining regular physical activity is challenging for most people, as many struggle to meet the recommended 150 min of moderate-intensity exercise per week [50]. Individuals with severe mental illness are 50% more likely to fall short of these guidelines than the general population [51] and show low long-term adherence to physical activity interventions [10]. Consistent with these findings, the present study suggests that sustaining physical activity after psychiatric treatment was perceived to be a key challenge that warrants further attention. Although participants expressed intentions to remain active, few reported concrete plans or confidence in maintaining activity on their own in the future. This gap, evident in both interviews and questionnaire data, supports previous research indicating systemic weaknesses in community follow-up [38, 52].

Sustained engagement in physical activity after psychiatric treatment has received limited focus in research [38, 49]. In our study, participants perceived existing follow-up as vague and insufficient, reinforcing concerns that current strategies are far from optimal [16]. Findings from both methods indicated that participants wanted to continue with the same type and structure of activity offered during hospital treatment. However, the lack of comparable community programs may reduce motivation and create uncertainty about how to continue independently [16]. Overall, these findings point to the need for closer collaboration between specialist services, municipalities, and primary care. A stronger link between structured inpatient programs and community-based opportunities may help ensure a smoother transition and better long-term maintenance of physical activity for individuals with severe mental illness.

### Strengths and limitations

The key strength of this study lies in its mixed-methods convergent design, which allowed for the integration of qualitative and quantitative data to corroborate and enrich the findings. Conducting the research in a real-world clinical setting strengthened ecological validity and provided insights relevant for both clinical practice and policy development. The study emphasized ethical considerations, participant voluntariness, and safety, which are essential when conducting research involving individuals with severe mental illness. Another strength was the timing of the interviews, conducted immediately after the activity sessions, which helped capture participants’ spontaneous and authentic reflections.

Some limitations should be considered. Recruitment bias may have favored individuals with positive attitudes toward physical activity and underrepresented those who were less engaged or had more severe symptoms, comorbidities, bipolar disorder, or major depressive disorder. As a result, potential negative experiences from individuals who declined or discontinued participation in physical activity were not captured, and the findings primarily reflect the views of those already willing to engage in the program. Consequently, the results may not represent the broader population of people with severe mental illness. Additionally, because SEH was a familiar figure to some participants, this may have increased trust and openness. However, it may also have introduced social desirability bias, leading participants to feel obligated to take part or to give affirmative responses. While the timing of the interviews allowed for immediate reflection, it may also have introduced fatigue or “primed” participants through the positive experience of having completed an activity. Interviews were conducted and transcribed in Norwegian, and specific quotations were translated into English, which may have resulted in a minor loss of cultural nuance. Furthermore, neither the instruments used to develop the questionnaire nor the final version has been validated for individuals with severe mental illness, and several items generated simple frequency data, limiting the depth of quantitative interpretation. Finally, the single-site, cross-sectional design limits both generalizability and insight into long-term effects.

### Future research and practical implications: towards a more active mental health care

In line with recent research [6, 35], the findings of this study underscore the importance of integrating physical activity as a patient-centered, standard component of psychiatric care. The Diakonhjemmet Hospital activity program could serve as a model for adaptation to other contexts. Future studies should explore diagnostic differences, reasons for non-participation, and sustained engagement through longitudinal designs.

Developing structured transition plans in collaboration with patients and community organizations appears to be crucial for maintaining continuity from inpatient to community-based activity. Mental health services should also include professionals such as exercise physiologists, skilled in the therapeutic use of physical activity and individualized motivational support.

The main challenge is no longer the lack of evidence but the implementation gap. Embedding physical activity into psychiatric treatment will require a national commitment, including investment in infrastructure, staffing, and funding to ensure that exercise becomes a systematic, integrated, and supported element of mental health care.

## Conclusions

Participants valued the weekly group-based physical activity program for fostering well-being, social connection, purpose, and structure. However, barriers such as medication side effects and fluctuating motivation often hindered participation. Supportive and competent activity leaders played a key role in addressing these barriers and encouraging sustained engagement. Despite intentions to remain active after treatment, participants perceived current follow-up support as limited and inconsistent. The study’s real-world context and person-centered approach support the need for more research to evaluate best practices, then implement and sustain strategies to ensure that individuals with severe mental illness have genuine opportunities to establish physical activity as a routine part of daily life.

## Supplementary Information

Below is the link to the electronic supplementary material.

Supplementary Material 1: Additional file 1 (file: .pdf. Title: Interview guide. Description: Interview guide for the semi-structured interviews in English translated version from Norwegian).

Supplementary Material 2: Additional file 2 (file: .pdf. Title: The questionnaire. Description: the distributed paper-based questionnaire in English translated version from Norwegian).

Supplementary Material 3: Additional file 3 (file: .pdf. Title: Participant descriptives of the interviewees. Description: characteristics of the interviewed participants (n = 8) (gender, age category in years, physical activity in treatment in days per week, and type of activity, physical activity outside of treatment yes/no, and type of activity, and mental wellbeing on the day of data collection).

Supplementary Material 4: Additional file 4 (file: .pdf. Title: Quantitative data. Description: descriptive statistics of the questionnaire data related to each study area presented in tables S1, S2, and S3.

## Data Availability

All data generated or analyzed during this study are included in this published article (and its supplementary information files), except for the raw interview data, due to the privacy of vulnerable participants. However, anonymized excerpts or aggregated data may be available from the corresponding author upon reasonable request.

## Abbreviations

MiM: Mind in Motion
NIH: Norges idrettshøgskole [Norwegian School of Sports Science
SD: Standard deviation
BMI: Body mass index
n: Number
COPD: Chronic obstructive pulmonary disease
PA: Physical activity
