# Supplementary material for: Mind in motion: patients’ experiences with group-based physical activity in psychiatric treatment- a mixed-methods study
Source: BMC Psychiatry. 2026 Apr 26;26:461. doi: 10.1186/s12888-026-08117-7 (PMC13267368; doi:10.1186/s12888-026-08117-7)
Supplement: Supplementary file 1 — Supplementary Material 1: Additional file 1 (file: .pdf. Title: Interview guide. Description: Interview guide for the semi-structured interviews in English translated version from Norwegian). [file 12888_2026_8117_MOESM1_ESM.pdf]

Additional file 1: Interview guide (English translation)

Interview Guide- Physical Activity in Psychiatric Treatment

With patients from the specialized outpatient clinic, Diakonhjemmet Hospital, Department of Adult Psychiatry, Vinderen

Tentative research question:

- *What perspectives and experiences do patients from the specialized outpatient clinic at Diakonhjemmet Hospital have regarding participation in group-based physical activity as part of their psychiatric treatment?*

<u>Study-foci and research questions to cover:</u>

- Perceived health benefits of PA:
  - What are the perceived physical, mental, and social health benefits of engaging in weekly group-based PA?
- Perceived barriers, challenges, and possible management strategies:
  - What are specific barriers that patients commonly encounter when participating in weekly PA, and how could these be minimized according to the patients?
- Sustained engagement in PA:
  - What factors may contribute to sustained engagement in PA beyond the duration of formal psychiatric treatment?

Introduction and **information** about the interview/project:

- *Introduce myself, my role, and why I am here*

My name is Siri, and I am a master's student at the Norwegian School of Sport Sciences. I am writing my master's thesis on physical activity and mental health. I previously had a placement at Diakonhjemmet, where I was allowed to observe and learn about the activity program that you are participating in.

- *Information about the MiM project and its purpose + thank participant*

Thank you so much for agreeing to participate in this project. I really appreciate it. The purpose of the project is to explore experiences and perceptions related to physical activity in mental health care. Your perspectives are important for improving this. You will first complete a questionnaire (approx. 5–10 minutes), and then we will conduct an interview of about 45 minutes.

- *Information about voluntary participation, informed consent, and project approval*

You have previously signed a consent form (show the form), meaning you have voluntarily agreed to participate in the research project. Participation is always voluntary, and you may withdraw at any time without any consequences. The project has been ethically approved.

- *Information about the interview (themes, duration), audio recording, de-identification, storage of information*

The interview will focus on your experiences and perceptions of participating in the weekly physical activity program at Diakonhjemmet. The themes we will discuss include perceived benefits and health effects, challenges that may arise, and physical activity after treatment ends. The interview will be audio-recorded, transcribed verbatim, and then the audio file will be deleted. You will remain de-identified throughout the entire project, and all collected information will be securely stored at NIH.

- *Information about participant autonomy*

You decide what you wish to answer and talk about, and you may pause or withdraw at any time without giving a reason. There are no right or wrong answers, so please be honest and answer as best you can.

Do you have any questions before we begin?

<u>*Completion of the questionnaire:*</u>
You will first fill out a questionnaire. If you have any questions or if something is unclear, please feel free to ask.
Then we will continue with the interview. Some of the questions in the questionnaire and interview may be quite similar; this is intentional.

Inform the participant that the audio recording is starting.

- **Activity habits-** building rapport and safety, "easy" start
  a. Can you please tell me a bit about your relationship with physical activity and exercise *before* joining the activity program at Diakonhjemmet?
     - *What activities did you do as a child? Was your family active, and what did you do together?*
     - *What did you think about PE in school? Did you participate in organized sports? What types of activities did you do as a teenager/young adult?*
  b. How long have you been participating in the Diakonhjemmet activity program?
     - *When did you start treatment here, and how long was it before you were offered the opportunity to participate in the physical activity program?*
  c. Which activities in the program do you usually participate in during a week?
     - *In winter? And in spring, summer, and autumn?*

- **Perceived benefits, health effects, and challenges/barriers**
  a. How do you experience participating in the activity/activities? How do you feel about being active in a group with others?
     - *How do you feel before, during, and after the activity? Why do you think that is? Too hard/too easy?*
     - *Do you primarily see the program as a social offer or as an exercise/activity program?*
     - *The program is group-based… what are the advantages and disadvantages? Is there too little/too much focus on the social aspects?*
  b. What are your main reasons for participating in the activity/activities?
     - *Why did you choose to join? What does it give you? What motivates you the most?*

  c. Since joining the weekly activity program, do you feel anything has changed:
     - Physically/bodily- in what way?
       - *How has physical activity affected your body? (for example, muscle strength, endurance/fitness, balance/coordination, sleep, less illness/improved immune function)*
     - Mentally/psychologically- in what way?
       - *Do you have more energy, feel less stress, fewer symptoms and difficult periods, a more stable/better mood, a sense of distraction, increased self-efficacy, improved self-image and self-confidence, a sense of meaning, something to do, or that you handle challenges better, etc. Why do you think that is?*

- Socially- in what way?
  - *New acquaintances/friends, support from others, understanding of others and feeling understood, improved social functioning, increased independence, a sense of belonging and togetherness, etc. In what way do you feel this? Why do you think that is?*

d. What challenges can you experience related to participating in the physical activity program? (before, during, after).
   - *What might prevent you from taking part in an activity?*
   - *What do you find difficult or challenging during the activity itself?*
   - *Is there anything challenging that can happen after you have participated in the activity?*
   - *Is there anything you find difficult about taking part in the activity program in general?*

e. Can you tell me about a specific day when you planned to go but didn't?
   - *Can you recall a day when you were unable to go to the activity you had planned to attend? What happened then? Why do you think it turned out that way? How did you feel about it?*

f. Considering the barriers and challenges we just discussed, is there anything you think could make it easier for you to participate in the activity program?
   - *Do you have any suggestions for changes that might make it easier to take part in the activities (before and/or during them)? Is there anything you feel is missing, or anything you would like more or less of?*

- **Maintaining physical activity after treatment**
  a. If you imagine a situation where you are no longer in treatment here… do you want to stay physically active?
     - *In what way / why not?*
     - *If yes: what kinds of activities would you like to do?*
     - *What do you think you would need in order to manage that?*
     - *Are you aware of other available opportunities or activity options?*

- **Ending the interview**
  a. Is there anything else you would like to talk about that I haven't asked about?
  b. Do you have any questions for me?
  c. Thank you for participating.

The questions in italics: Follow-up questions in cases of short answers or when elaboration is needed.
