# Supplementary material for: Mind in motion: patients’ experiences with group-based physical activity in psychiatric treatment- a mixed-methods study
Source: BMC Psychiatry. 2026 Apr 26;26:461. doi: 10.1186/s12888-026-08117-7 (PMC13267368; doi:10.1186/s12888-026-08117-7)
Supplement: Supplementary file 2 — Supplementary Material 2: Additional file 2 (file: .pdf. Title: The questionnaire. Description: the distributed paper-based questionnaire in English translated version from Norwegian). [file 12888_2026_8117_MOESM2_ESM.pdf]

Additional file 2: The questionnaire (English translation)

Dear participant,

We know that physical activity has a positive effect on our mental health. However, there is still little knowledge about how this can be used in the treatment of mental disorders and how this is experienced by the participants.

By answering this questionnaire, you are contributing to important knowledge. The questionnaire takes about 10 minutes to complete and will be de-identified.

Please be honest; there are no right or wrong answers. Some of the questions may seem similar, which is intentional, so please read carefully.

Select the answers that best apply to you and check the box.

Thank you very much for your help 😊

Activity habits:
We would like to know about your physical activity habits both before and after you joined the physical activity program offered by the resource team at Vinderen.

Think back to the time ***before*** you joined the activity program at Vinderen...

1. On average, how many times a week did you engage in physical activity (all forms, including exercise/sports) that made you sweat a little and breathe a little more than usual (moderate-hard intensity)?
   - ❑ 0 times per week
   - ❑ 1 time per week
   - ❑ 2 times per week
   - ❑ 3 times per week
   - ❑ 4 times per week
   - ❑ 5 times per week
   - ❑ 6 times per week
   - ❑ Every day

2. How long did this activity last on average each time?
   - ❑ Was not physically active
   - ❑ Less than 30 minutes
   - ❑ 30-60 minutes
   - ❑ 61-90 minutes
   - ❑ Over 90 minutes

3. What kind of physical activity did you usually engage in *before* joining the activity program offered by the resource team at Vinderen? You can check multiple boxes.
   - ❑ Active commuting (walking, cycling, etc., to/from work/school)
   - ❑ Activities at home (e.g., housework and gardening)
   - ❑ Going for walks/hiking
   - ❑ Cycling
   - ❑ Working out at the gym (all types)
   - ❑ Sports clubs/sports halls (handball, soccer, squash, cross-country skiing, swimming, etc.)
   - ❑ Exercising at home
   - ❑ Other
   - ❑ Was not physically active

4. How long have you participated in the physical activity program offered by the resource team at Vinderen?

Answer: ____

5. How long were you in treatment at Diakonhjemmet *before* you found out about the physical activity program?

Answer: ____

6. Which activities organized by the resource team at Vinderen do you usually participate in during a week? Check the box(es):
   - ❑ Hiking (walking, running)
   - ❑ Strength/circuit training
   - ❑ Cross-country skiing in winter
   - ❑ Swimming
   - ❑ Soccer (including «gatelaget»)
   - ❑ Volleyball

7. Are you physically active outside of the physical activity program offered by the resource team?
   - ❑ Yes
   - ❑ No

8. If you answered YES to the previous question (question 7): What kind of activities do you engage in outside of the activities offered by the resource team?
   - ❑ Active commuting (walking, cycling, etc., to/from work/school)
   - ❑ Activities at home (e.g., housework and gardening)
   - ❑ Going for walks/hiking
   - ❑ Cycling
   - ❑ Working out at the gym (all types)
   - ❑ Sports clubs/sports halls (handball, soccer, squash, cross-country skiing, swimming, etc.)
   - ❑ Exercising at home
   - ❑ Other

Experiences with the activity program:
We would like to know how you feel about participating in the physical activity program offered by the resource team at Vinderen.

9. Below are a number of reasons for participating in the activities offered by the resource team. Please tick one or more boxes for the reason(s) that are important to you.

   a. Physical reasons
      - ❑ Prevent health problems
      - ❑ To have a healthy body/good health
      - ❑ To get in better shape
      - ❑ Maintain weight
      - ❑ To look fit

   b. Psychological reasons
      - ❑ Get fresh air/get outside
      - ❑ Experience excitement/challenge
      - ❑ Is a form of distraction
      - ❑ Have fun
      - ❑ I feel like I have to
      - ❑ Deal with stress

   c. Social reasons
      - ❑ To meet and socialize with other people
      - ❑ To be with others who have similar mental health issues
      - ❑ The activity leaders motivate me to participate
      - ❑ Recommended by a doctor/therapist/health care professional

Other reasons, what:____

10. Do you feel that participating in the activities offered by the resource team at Vinderen has had a positive effect on your:

Physical fitness and health? (e.g. better endurance/less fatigue, increased strength, improved motor skills/balance/coordination, fewer physical ailments and pains, better sleep):

❍ 10	❍ 9	❍ 8	❍ 7	❍ 6	❍ 5	❍ 4	❍ 6	❍ 2	❍ 1
Very positive effect									No positive effect

Mental health and well-being? (e.g. more energy, fewer symptoms, less stress, better self-esteem/confidence, concentration, sense of mastery):

❍ 10 Very positive effect	❍ 9	❍ 8	❍ 7	❍ 6	❍ 5	❍ 4	❍ 6	❍ 2	❍ 1 No positive effect

11. Participation in the physical activity program has helped to reduce self-destructive behavior/poor habits (e.g., drug use- including alcohol, poor sleep patterns, self-harm, overeating/starvation, isolation).
    - ❑ Yes
    - ❑ No
    - ❑ Not relevant to me

12. Do you think that adapted physical activity should be a permanent part of psychiatric treatment/mental health care?
    - ❑ Strongly agree
    - ❑ Agree
    - ❑ Neither agree nor disagree
    - ❑ Disagree
    - ❑ Strongly disagree

13. I feel supported by the activity leaders in the resource team
    - ❑ Strongly agree
    - ❑ Agree
    - ❑ Neither agree nor disagree
    - ❑ Disagree
    - ❑ Strongly disagree

14. How do you feel about being active with others in a group compared to being active on your own?
    a. It is more motivating:
        - ❑ Agree
        - ❑ Disagree
        - ❑ No difference

b. It makes me more committed, and I show up more often:
   - ❑ Agree
   - ❑ Disagree
   - ❑ No difference

c. It makes me function better socially:
   - ❑ Agree
   - ❑ Disagree
   - ❑ No difference

15. Think back over the last month (last 4 weeks), how many times on average per week <u>did you find yourself unable to participate</u> in planned activities organized by the resource team at Vinderen (for whatever reason)?
    - ❑ 0 times per week
    - ❑ 1 time per week
    - ❑ 2 times per week
    - ❑ 3 times per week
    - ❑ 4 times per week
    - ❑ 5 times per week

16. What are the typical reasons why you are <u>*unable*</u> to participate in activities organized by the resource team? Feel free to check more than one box.

    a. <u>Practical barriers</u>
    - ❑ Appointment with a health care professional at the outpatient clinic
    - ❑ Transportation
    - ❑ Do not have the necessary equipment/clothing
    - ❑ Weather
    - ❑ Work
    - ❑ Family obligations

    b. <u>Prioritization barriers</u>
    - ❑ Don't have time
    - ❑ Lack of energy
    - ❑ I want to do other things

    c. <u>Emotional/cognitive barriers</u>
    - ❑ Forget about it
    - ❑ Don't think I can do the activity
    - ❑ Don't like the activity in question
    - ❑ Anxious about going out

❑ Social discomfort in the exercise group
❑ Can't be bothered/tired/unmotivated

d. Health barriers
❑ Due to my physical health/mobility problems
❑ Illness/not in shape
❑ I don't think it's important for my own health

Other reasons, what:______________________________________

Maintaining physical activity levels after completing treatment:
We want to know what you think about physical activity when you are discharged from treatment at Diakonhjemmet.

Imagine a time when you are *no* longer undergoing treatment at Diakonhjemmet...

17. Do you want to continue being physically active?
    ❑ Yes
    ❑ No
    ❑ Not sure

18. Do you think you will manage to stay physically active?
    ❑ Yes
    ❑ No
    ❑ Don't know

19. What activities would you like to do after completing treatment at Diakonhjemmet? (feel free to check more than one box)

❑ Hiking/walking
❑ Dancing
❑ Golf
❑ Cross-country skiing
❑ Yoga/Pilates
❑ Tennis
❑ Strength training
❑ Ball games
❑ Swimming
❑ Water aerobics
❑ Other
❑ Alpine skiing/snowboarding
❑ Martial arts (karate, judo, etc.)
❑ Canoeing/rowing
❑ Cycling/spinning
❑ Jogging/running
❑ Skating/bandy/hockey
❑ Exercising to music
❑ Squash/Badminton/Table tennis

20. How many times a week do you want to be physically active after completing treatment at Diakonhjemmet?
    - ❑ 0 times per week
    - ❑ 1 time per week
    - ❑ 2 times per week
    - ❑ 3 times per week
    - ❑ 4 times per week
    - ❑ 5 times per week
    - ❑ 6 times per week
    - ❑ Every day

21. Would you like to continue being active with others or on your own?
    - ❑ Active with others/in a group
    - ❑ Active alone
    - ❑ Both

22. Are you aware of other opportunities/offers for physical activity that you can join after being discharged from Diakonhjemmet?
    - ❑ Yes
    - ❑ No

Background/health:

Gender:

- ❑ Male
- ❑ Female
- ❑ I do not identify as male or female

Age (years): ____________

Height (cm): ___________

Weight (kg): _____________

What is your highest level of education?

- ❑ Elementary school (primary and secondary school)
- ❑ High school
- ❑ College/university
- ❑ Other education

What is your current employment status:

- ❑ Working 50% or more
- ❑ Working 50% or less
- ❑ Not working/disabled
- ❑ Student/apprentice

Has your doctor diagnosed you with: (feel free to check more than one box)

- ❑ Asthma
- ❑ Allergy
- ❑ Chronic bronchitis/emphysema/COPD
- ❑ Heart attack
- ❑ Angina pectoris (heart cramps)
- ❑ Stroke/cerebral hemorrhage (drip)
- ❑ Type I diabetes (diabetes mellitus)
- ❑ Type II diabetes (diabetes mellitus)
- ❑ Hypertension (high blood pressure)
- ❑ Osteoporosis
- ❑ Cancer
- ❑ Eating disorder
- ❑ Rheumatic disorder
- ❑ None of these

I think I am...

- ❑ Underweight
- ❑ Normal weight
- ❑ Overweight
- ❑ Fat
- ❑ Do not wish to answer

Psychiatric diagnosis:

- ❑ Bipolar disorder
- ❑ Schizophrenia
- ❑ Other psychotic disorder
- ❑ Do not wish to answer

Current mood: Set an X on the line below to indicate how you feel **TODAY**.

0 — 1 — 2 — 3 — 4 — 5 — 6 — 7 — 8 — 9 — 10

0= Worst possible mental well-being

10= Best possible mental well-being

Thank you for participating! ☺
