# Supplementary material for: Mind in motion: patients’ experiences with group-based physical activity in psychiatric treatment- a mixed-methods study
Source: BMC Psychiatry. 2026 Apr 26;26:461. doi: 10.1186/s12888-026-08117-7 (PMC13267368; doi:10.1186/s12888-026-08117-7)
Supplement: Supplementary file 3 — Supplementary Material 3: Additional file 3 (file: .pdf. Title: Participant descriptives of the interviewees. Description: characteristics of the interviewed participants (n = 8) (gender, age category in years, physical activity in treatment in days per week, and type of activity, physical activity outside of treatment yes/no, and type of activity, and mental wellbeing on the day of data collection). [file 12888_2026_8117_MOESM3_ESM.docx]

Additional file 3: Participant descriptives of the interviewees

In-depth interviewed participants

*Table 1: Characteristics of the interviewed participants (n=8).*

| **Name**  (pseudonym) | ***Anna*** | ***Mary*** | ***Daniel*** | ***Helen*** | ***Sophia*** | ***Eric*** | ***Linda*** | ***John*** |
| --- | --- | --- | --- | --- | --- | --- | --- | --- |
| Gender | Female | Female | Male | Female | Female | Male | Female | Male |
| Age category  (years) | 30-39 | 60-69 | 30-39 | 50-59 | 40-49 | 30-39 | 20-29 | 60-69 |
| PA^1^ in  treatment (days per  week + type of activity) | Two days:   - Hiking - Strength- training | Two days:   - Hiking - Swimming | Four days:   - Hiking - Cross- country skiing - Swimming - Football | One day:  - Hiking | Four days:   - Hiking - Cross- country skiing - Swimming - Volleyball | Two days:   - Hiking - Swimming | One day:  - Swimming | Two days:   - Hiking - Swimming |
| PA outside of treatment  (yes/no + type of activity) | Yes:   - Active commuting^2^ - Hiking | No | Yes:   - Active at home - Other   activity | Yes:   - Hiking - Gym | No | Yes:   - Active commuting^2^ - Hiking - Biking | Yes:  - Active commuting^2^ | Yes:  - Hiking |
| Mental wellbeing  (0-10)^3^ | 3.0 | 7.0 | 7.0 | 9.0 | 10.0 | 10.0 | 7.5 | 10.0 |

Note: ^1^PA= physical activity, ^2^Active commuting= physical activity as means of transportation, for example walking or using a bicycle, ^3^Perceived mental state on the day of the interview (scale of 0-10: 0= worst imaginable psychological well-being and 10= best imaginable psychological well-being).

Table 1 does not include specific psychiatric diagnoses to maintain confidentiality, however over half of the participants had a schizophrenia spectrum disorder. None were employed at the time. To further maintain confidentiality, all eight participants were assigned randomly generated fictional names based on gender and placed in age categories.
