# Supplementary material for: Mind in motion: patients’ experiences with group-based physical activity in psychiatric treatment- a mixed-methods study
Source: BMC Psychiatry. 2026 Apr 26;26:461. doi: 10.1186/s12888-026-08117-7 (PMC13267368; doi:10.1186/s12888-026-08117-7)
Supplement: Supplementary file 4 — Supplementary Material 4: Additional file 4 (file: .pdf. Title: Quantitative data. Description: descriptive statistics of the questionnaire data related to each study area presented in tables S1, S2, and S3. [file 12888_2026_8117_MOESM4_ESM.docx]

Additional file 4: Quantitative data

Table S1: Questionnaire data (n=33) related to perceived health benefits of physical activity. Variables are presented as number and percentage (n (%)) or mean and standard deviation (mean (SD)).

| **Variables** |  | **Total sample (n=33)** |
| --- | --- | --- |
| Physical health-related factors for PA^1^ participation (n (%)): | |  |
| Have a healthy body | | 30 (90.9) |
| Get in better shape | | 24 (72.7) |
| Preventing health problems | | 20 (60.6) |
| Keep the weight in check | | 19 (57.6) |
| To look fit | | 7 (21.2) |
| Psychological health-related factors for PA participation (n (%)): | |  |
| To get fresh air | | 30 (90.9) |
| It’s fun | | 16 (48.5) |
| It’s a distraction | | 14 (42.4) |
| To handle stress | | 11 (33.3) |
| I feel like I must | | 7 (21.2) |
| To experience excitement | | 5 (15.2) |
| Social health-related factors for PA participation (n (%)): |  |  |
| Meeting other people |  | 27 (81.8) |
| The activity leaders motivate me |  | 20 (60.6) |
| Recommended by a health professional |  | 18 (54.5) |
| To be with others in similar situations |  | 8 (24.2) |
| Perceived positive effect of PA on physical health (1-10) (mean (SD) | | 7.9 (1.5) |
| Perceived positive effect of PA on mental health (1-10) (mean (SD) | | 7.7 (2.1) |
| Reduction of destructive behavior (such as |  |  |
| substance abuse, self-harm, isolation) (n (%)) |  |  |
| Yes: |  | 17 (51.5) |
| Not relevant for me: |  | 14 (42.4) |
| Effects of the PA group (n (%)): | Agree: | No difference: |
| It’s more motivating | 27 (81.8) | 6 (18.2) |
| It makes me participate more often | 24 (72.7) | 9 (27.3) |
| It makes me function better socially | 24 (72.7) | 9 (27.3) |

*Note*: ^1^PA*=* physical activity, ^2^Perceived effect on physical/mental health (scale 1-10: 1= no positive effect, 10= very positive effect)

Table S2: Questionnaire data (n=33) related to perceived barriers and possible management strategies. Variables are presented as number and percentage (n (%)) or mean and standard deviation (mean (SD)).

| **Variables** | **Total sample (n=33)** |
| --- | --- |
| Days in the last four weeks unable to participate in a specific activity due to various reasons (such as illness, injury, or other  commitments) (mean (SD)) | 0.9 (0.9) |
| Barriers to PA participation (n (%))  Another appointment for treatment Too tired  Want to do other things No energy to participate Sickness/ feeling unwell Weather  Transportation Work  Family commitments  Physical health issues/difficulty moving Don’t have time  I forgot  I don’t like the activity Afraid/anxious to go outside Social discomfort  Lack of equipment/ clothes | 17 (51.5)  16 (48.5)  15 (45.5)  12 (36.4)  11 (33.3)  8 (24.2)  6 (18.2)  5 (15.2)  5 (15.2)  4 (12.1)  3 (9.1)  3 (9.1)  1 (3.0)  1 (3.0)  1 (3.0)  1 (3.0) |
| I feel supported by the activity leaders (n (%)) Strongly agree:  Agree:  Neither agree nor disagree: Disagree:  Strongly disagree: | 29 (87.9)  3 (9.1)  1 (3.0)  0 (0)  0 (0) |
| PA should be a permanent part of mental health care (n (%)) Strongly agree:  Agree:  Neither agree nor disagree: Disagree:  Strongly disagree: | 23 (69.7)  9 (27.3)  1 (3.0)  0 (0)  0 (0) |

*Note:* PA*=* physical activity

Table S3: Questionnaire data (n=33) related to sustained engagement in physical activity. Variables are presented as number and percentage (n (%)) or mean and standard deviation (mean (SD)).

| **Variables** | **Total sample (n=33)** |
| --- | --- |
| Intention to continue PA after treatment (n (%)) Yes:  Do not know: | 32 (97.0)  1 (3.0) |
| Confidence in maintaining PA post-treatment (n (%)) Yes:  Do not know: | 27 (81.8)  6 (18.2) |
| Intended PA level after treatment (days per week) (mean (SD)) | 3.2 (1.6) |
| Preferred type of activity after treatment (n (%)) Hiking  Strength training Swimming Yoga/Pilates Jogging/running  Cross-country skiing Dance  Downhill skiing/Snowboarding Cycling/spinning  Ball games/sports Golf  Rowing  Bandy/Hockey/Ice-skating Aerobic/exercise to music  Racket sports (tennis/squash/badminton/table tennis) Other  Water gymnastics | 27 (81.8)  16 (48.5)  14 (42.49  12 (36.4)  10 (30.3)  9 (27.3)  7 (21.2)  6 (18.2)  6 (18.2)  4 (12.1)  2 (6.1)  2 (6.1)  2 (6.1)  3 (9.1)  2 (6.1)  2 (6.1)  1 (3.0) |
| Preference for exercising alone or with others after treatment (n (%)) Both  With others Alone | 25 (75.8)  5 (15.2)  3 (9.1) |
| Yes, I am familiar with other available PA options (n (%)) | 21 (63.6) |

*Note:* PA*=* physical activity
